# Impacts of knowledge and trust on consumer perceptions and purchase intentions towards genetically modified foods

**DOI:** 10.1371/journal.pone.0311257

**Published:** 2024-10-02

**Authors:** Thi Thuy An Ngo, Thi Yen Nhi Phan, Thi Ngoc Trang Le

**Affiliations:** 1 Department of Soft Skills, FPT University, Can Tho City, Vietnam; 2 Department of Business, FPT University, Can Tho City, Vietnam; Center for Research and Technology Transfer, VIET NAM

## Abstract

Genetically Modified Foods (GMF) have emerged as a significant topic within the global food industry, provoking extensive debates over their safety and impact on consumer choices. This research aims to explore the impact of knowledge and trust in GMF on Vietnamese consumers’ perceptions and their subsequent purchase intentions. By employing a quantitative methodology, this study gathered data from 424 valid respondents. The research model focuses on five constructs: knowledge, trust, perceived benefits, perceived risks, and purchase intentions. The data was analyzed using Partial Least Squares Structural Equation Modeling (PLS-SEM) to test hypotheses and examine the interrelationships among these constructs. The results showed that increased knowledge of GMF is linked to heightened perceptions of benefits and reduced perceptions of risks, thereby elevating purchase intentions. Trust in GMF significantly enhances perceived benefits but does not significantly affect risk evaluation, although it is positively correlated with purchase intentions. Moreover, perceptions of greater benefits are positively associated with higher purchase intentions, whereas increased risk perceptions negatively influence them. These results underscore the complex relationship among knowledge, trust, perceived benefits, and perceived risks in shaping consumer purchase intentions toward GMF. The study proposes a comprehensive model delineating how knowledge and trust impact Vietnamese consumers’ perceptions and purchase intentions regarding GMF. These findings provide implications for policymakers, business managers, and marketers, emphasizing the importance of disseminating transparent information, prioritizing trust-building, and adopting nuanced communication strategies. By effectively enhancing perceived benefits and addressing perceived risks, these strategies aim to foster positive consumer intentions and behaviors toward GMF.

## 1. Introduction

Nowadays, food insecurity is a significant issue in both developed and developing countries. In developed countries, food insecurity is often associated with inequality and inadequate social protections, leading to a reliance on food banks and food waste diversion efforts [1]. On the other hand, developing countries face challenges such as low food production, postharvest losses, and acute hunger [2]. Addressing this issue, Thangarajan [3] posited that genetically modified foods (GMF) and crops hold promise for enhancing food security, fostering socio-economic improvements among farmers, and causing minimal adverse effects on the environment and human health.

Genetically modified organisms (GMOs) involve organisms, including plants or animals, that have been altered using gene technology to combine genes from different organisms [4]. The Food and Agriculture Organization of the United Nations reports that over 17 million farmers in 29 countries cultivate genetically modified crops, which are yielding better results with reduced pesticide usage, improved weed control, and various other advantages [5]. GMF has gained popularity worldwide, with the US being a major consumer of genetically modified (GM) crops such as corn and soybeans [6].

The potential advantages associated with GM crops include improvements in agricultural productivity, particularly through the development of crops resistant to insects and drought, thereby mitigating global hunger [7]. Rodríguez [8] asserted that GMF offers significant economic, environmental, food security, and human health benefits. Thangarajan [3] suggested that GMF and crops may alleviate the challenge of insufficient food production to support the growing population. The positive impacts extend to improvements in food security, poverty reduction, and disease prevention [9]. Moreover, Boccia and Punzo [10] found that consumers perceive and appreciate the benefits of GMF, including improved nutritional qualities and health benefits.

Consumer knowledge of GMF influences their perceived benefits and risks in various ways. While studies indicate that consumers’ knowledge influences these perceptions, the impact of knowledge on reducing perceived risks is not consistently significant [11]. Additionally, McPhetres [12] reported that acquiring knowledge about the science behind GM technology leads to more favorable attitudes, increased willingness to consume GM products, and a decreased perception of risks. However, individuals with higher education, income, and food involvement, when exposed to negative information about GMF, tend to overestimate their knowledge, resulting in increased risk perception, decreased benefit perception, and lower intent to buy such foods [13]. The study by Rodríguez [8] pointed out that consumer knowledge about GMF is limited, and individuals rely on trustworthy experts to form their views on the benefits and risks.

Nevertheless, the research findings indicated that consumers exhibit both negative and positive attitudes toward GMF, with social equity, trust, and health concerns playing a significant role in attitude formation [14]. According to Deng and Hu [15], consumer acceptance of GMF is positively influenced by trust in GM scientists and the government, while distrust in non-GM scientists or individuals, along with belief in misinformation, negatively affects acceptance. In Brazil, most consumers do not recognize the symbol used in mandatory labeling of GMF, but those who do have a high confidence in science and the government, reducing their risk perception [16]. Good media coverage positively impacts people’s trust in the government and belief in science, which may impact the perception of GMF [17].

In recent years, researchers have identified various factors impacting consumers’ perceptions and their intention to purchase GMF. Low-income countries exhibit higher support for GMF compared to affluent nations, with individual attitudes shaped by living standards, agricultural output, and the prevalence of undernourishment [18]. A recent study on Chinese consumers revealed that factors such as awareness, product availability specifics, seller trust, product taste, and purchasing experience significantly impact their willingness to purchase GMF [19]. Meanwhile, research conducted in Jordan reveals that the determinants of purchase intention for GMF involve relative advantage, compatibility, trialability, social approval, awareness, perceived risk, and price value [20]. According to Aliasgharzadeh [21], willingness to purchase GMF is adversely affected by knowledge, whereas trust in GM institutes positively determines the inclination to buy. Among these factors, knowledge and trust in GMF are crucial and influential components.

In developing countries like Vietnam, where the concept of GMF is relatively new and their market presence is not as widespread or well known as in other countries, recent research has begun to explore the economic and environmental impacts of GM crops, especially focus on GM corn. Brookes and Dinh [22] conducted a study examining yield, production costs, and environmental effects. Pham and Napasintuwong [23] delved into the economic implications of adopting stacked Bt/Ht GM maize in Southern Vietnam, revealing significant benefits in terms of increased yield, revenue, and profit compared to non-GM counterparts. However, there has been a lack of exploration into factors such as knowledge, trust, perceptions about GMF, and consumers’ adoption and purchasing intentions in Vietnam. Therefore, a comprehensive investigation into how consumers’ knowledge and trust in GMF influence their purchasing intention becomes crucial. This study aims to fill the research gap by investigating consumers’ knowledge and trust levels regarding GMF in Vietnam and their impacts on purchasing intention, considering both direct and indirect pathways through perceived benefits and risks. The findings of the study can provide important information to GMF manufacturers, businesses, as well as state management agencies, helping them better understand consumer attitudes and behavior.

## 2. Literature review and hypotheses development

### 2.1. Theoretical background of genetically modified organisms (GMOs) and genetically modified foods (GMF)

In recent decades, the discourse surrounding the concepts of genetically modified organisms (GMOs) and genetically modified foods (GMF) has become highly contentious [24]. The term GMOs encompasses organisms excluding humans and refers to those deliberately altered in ways that deviate from natural processes such as mating or recombination [25]. The realm of GMF primarily involves plants, including soybeans, maize, and rapeseed, as well as other crops like potatoes, tomatoes, cotton, and tobacco. Additionally, there is a more limited inclusion of animals, notably cattle and pigs, along with microbes [26].

The utilization of GMF offers a range of multifaceted benefits. These benefits include addressing global hunger, creating superfoods fortified with additional vitamins and nutrients, and contributing to economic growth [27]. However, despite these advantages, consumers often express concerns about the potential risks associated with genetic modifications. These concerns primarily involve the impacts of molecular biology techniques that, by interfering with the natural recombination process, may disrupt the normal propagation abilities of organisms [26]. As a result, GMF products approved for global trade are subjected to comprehensive testing to scrutinize potential effects arising from consumption [28]. This rigorous evaluation aims to ensure the safety of GMF and alleviate public concerns. The intricate balance between potential benefits and perceived risks underscores the complexity of the debate surrounding GMF and GMO. This debate requires a nuanced examination of scientific, ethical, and societal dimensions. On one hand, there is the promise of technological advancements to solve critical issues like malnutrition and food scarcity. On the other hand, there are ethical considerations and potential ecological risks that need to be addressed.

The ongoing discourse reflects a broader societal dilemma: balancing innovation with precaution. The complexities involved in the adoption and regulation of GMF necessitate continuous research and dialogue among scientists, policymakers, and the public to navigate the potential benefits and risks effectively.

### 2.2. Knowledge of GMF (KN)

Consumer knowledge plays a crucial role in shaping behavior and purchase decisions regarding genetically modified foods (GMF). This knowledge is intricately linked to the trust consumers place in the information they receive, significantly influenced by the sources through which this information is disseminated [29]. The level of knowledge consumers possess about GMF directly affects their attitudes and, consequently, their buying behaviors. An informed consumer is more likely to make decisions based on a comprehensive understanding of the potential benefits and risks associated with GMF [30]. However, a notable challenge in this domain is the general lack of consumer knowledge about GMF. This knowledge gap can lead to skepticism and negative perceptions, often stemming from inadequate or misleading information. Such misinformation can overshadow the potentially significant implications and benefits of GMF, including enhanced nutritional content and agricultural efficiency [31]. In an era where information is readily accessible, the internet and media platforms play pivotal roles in shaping consumer knowledge about GMF. These channels provide a diverse range of information, from scientific studies to public opinions, which can significantly influence consumer attitudes. Among these sources, product labeling stands out as a crucial element, providing consumers with essential details about the nature and safety of GMF products [32, 33].

Research has shown that a comprehensive understanding of gene technology and specific knowledge related to GMOs significantly influences consumers’ perceptions regarding the benefits and risks associated with these organisms [34]. Notably, empirical evidence from Raza [35] demonstrated a discernible adverse impact of knowledge on the perceived risks associated with GMOs. This study suggests that improving individuals’ understanding of GMOs can alleviate concerns, indicating a direct inverse relationship between knowledge and perceived risk. In contrast, Ari [11] found that consumer knowledge levels significantly contribute to the augmentation of perceived benefits associated with GMOs. This implies that as consumers become more informed about the technological and scientific aspects of GMOs, they are more likely to recognize and appreciate the potential benefits, such as enhanced nutritional value and agricultural efficiency. These findings underscore the relationship between knowledge and both the perceived benefits and risks in the context of GMF.

The impact of consumer knowledge extends beyond perceptions of benefits and risks; it also significantly influences attitudes and purchase intentions toward GMF [13]. Enhanced knowledge leads to a more favorable attitude toward GMF and greater intention to purchase these products, as consumers who are well-informed are better equipped to evaluate the potential advantages and disadvantages [36]. This positive attitude, in turn, increases the likelihood of purchasing GMF. Andita [37] found that knowledge across various dimensions, such as the nature of GMF, its production processes, and its potential benefits, positively and significantly affects purchase intention. This finding underscores the critical role of consumer knowledge in shaping purchasing behavior. Similarly, Zhu [38] confirmed that consumers with extensive knowledge about GMF are more inclined to support and purchase these products. This correlation suggests that understanding the reasons behind and methods of GMF production fosters a more favorable consumer disposition. However, the relationship between knowledge and purchase intention is not always straightforward. Aliasgharzadeh [21] found that while positive attitudes toward GM technology and trust in GM institutes enhance the willingness to buy GMF, an increase in knowledge can sometimes have a negative impact. This counterintuitive finding suggests that greater knowledge might also make consumers more aware of potential controversies and ethical concerns, thus dampening their enthusiasm for GMF. Based on these insights, the hypotheses H1, H2 and H3 were proposed:

**H1:** Knowledge of GMF has a positive relationship with perceived benefits.
**H2:** Knowledge of GMF has a negative relationship with perceived risks.
**H3:** Knowledge of GMF has a positive relationship with purchase intention.

### 2.3. Trust on GMF (TR)

Trust is a multifaceted concept encompassing thoughts, feelings, emotions, and behaviors that emerge when customers believe a provider will act in their best interest, especially in situations where they relinquish direct control [39]. In the context of GMF, trust refers to the degree of belief that individuals have regarding the safety, reliability, and benefits of these products [40]. Trust is particularly crucial in influencing consumer attitudes toward GMF acceptance [41], as it encompasses confidence in the information provided by various entities, including the government, university scientists, and biotechnology researchers [32]. These sources are often viewed as impartial and reliable, contributing significantly to the overall perception of GMF.

Numerous studies consistently underscore the critical role of trust in shaping consumer perceptions of GMF, particularly in relation to the perceived benefits and risks. Lang [42] demonstrated that trust significantly affects perceptions of various risks associated with GMF, with increased social trust correlating with reduced apprehension about potential risks [43]. Moreover, Ari [11] extended this understanding by revealing that trust in scientists, the government, the labeling system, and the media enhances the perceived benefits of GMF. This indicates that trust not only positively influences perceived benefits but also mitigates perceived risks. Consumers who trust the entities responsible for GMF production and regulation are more likely to view these products favorably, acknowledging their potential benefits and downplaying possible risks [41]. Consequently, these collective insights establish trust as a key determinant in shaping both perceived benefits and risks.

The impact of trust extends beyond shaping consumer perceptions; it also plays a critical role in directly affecting consumers’ purchase intentions regarding GMF [44]. Lingyu and Liu [45] discovered a significant positive correlation between trust and purchase intention in the context of GM rice, indicating that consumers are more likely to purchase GM products when they trust the underlying technology and processes. This finding is consistent with a study by Akbari et al. [46], which found that when consumers trust the GM technology or the production process of a GM product, they exhibit positive intentions toward purchasing these products. Furthermore, Putra and Lestari [47] identified trust as a significant determinant of purchase intention, further emphasizing its pivotal role in shaping consumer attitudes and behaviors toward GM products. This body of evidence emphasizes the essential role of establishing and sustaining consumer trust to create a supportive market environment for GMF. Based on these findings, the hypotheses H4, H5 and H6 were proposed:

**H4:** Trust in GMF has a positive relationship with perceived benefits.
**H5:** Trust in GMF has a negative relationship with perceived risks.
**H6:** Trust in GMF has a positive relationship with purchase intentions.

### 2.4. Perceived benefits (PB) and perceived risks (PR)

GMF represents a pivotal advancement among transformative technological waves, holding immense potential to revolutionize food production by enhancing quality and nutritional value [48]. GMF technology holds promise in providing consumers with healthier and more nutritious options, incorporating added vitamins and nutrients into food products, thereby potentially mitigating food security threats, and fostering economic growth [27, 49]. However, despite the transformative potential of GMF in improving food production and nutritional value, apprehensions persist regarding potential health and environmental risks associated with GMF adoption. These risks include potential health issues, such as allergenicity and toxicity, as well as ecological risks like gene transfer and outcrossing [29, 50]. A study among Mexican educators illustrated this duality: while over 60% acknowledged the positive role of GMF in combating global hunger, 39.2% expressed concerns about the potential hazards they might pose to future generations [51].

The interplay between perceived benefits and perceived risks is critical in shaping consumer attitudes and behaviors towards GMF. Empirical investigation has demonstrated that perceived benefits play a crucial role in influencing consumers’ intent to purchase GMF. For example, Kim [52] underscored the significant role of perceived benefits in influencing consumer intent to purchase GMF. Another study also revealed that perceived risks associated with GMF collectively exert a negative influence on consumers’ purchase intentions toward GMF [41]. In addition, studies by Guo et al. [53], and Rodríguez-Entrena [54] have shown that heightened risk perceptions tend to reduce consumer propensity to purchase GMF, whereas acknowledging the benefits encourages a more favorable purchase intention. This nuanced relationship between perceived benefits, perceived risks, and purchase intentions underscores the importance of both factors in influencing consumer behavior. The insights from these studies support the development of hypotheses H7 and H8.

**H7:** Perceived benefits have a positive relationship with purchase intention.
**H8:** Perceived risks have a negative relationship with purchase intention.

### 2.5. Purchase intention (PI)

Purchase intention reflects the possibility, volition, plan, or willingness of consumers to buy a product or service in the future [55]. It serves as a critical indicator of consumer behavior and is influenced by a range of factors. In the context of GMF, several key determinants have been identified that significantly affect purchase intention.

One of the most influential factors is consumer knowledge. Research has demonstrated that a thorough understanding of GMF, including their benefits and risks, plays a crucial role in shaping purchase intentions [38]. Consumers who are well-informed about GMF are more likely to have a favorable view of these products and are consequently more inclined to purchase them.

Trust is another significant factor affecting purchase intention. It encompasses the degree of confidence consumers have in the safety, efficacy, and overall quality of GMF. Studies have shown that trust positively impacts purchase intention, as consumers are more likely to buy products from sources they trust [56]. This study also revealed positive moderating role of word-of-mouth (WOM) in the relationship between trust and consumer purchase intention. Moreover, Lingyu and Liu [45] found a significant positive correlation between trust and purchase intention concerning GMF rice. When consumers trust the technology or production process behind GMF, their intention to purchase these products increases.

Additionally, Additionally, research has indicated that consumer perceptions of risk and benefit also influence purchase intention. Participants who perceive higher risks associated with GMF, such as potential health or environmental impacts, often show lower purchase intentions. On the other hand, when perceived benefits, such as enhanced nutritional value or improved food security, are emphasized, consumers are more likely to express a willingness to purchase GMF products [13].

In summary, purchase intention towards GMF is shaped by a combination of factors, including consumer knowledge, trust, perceived benefits, and perceived risks. These factors interact in complex ways to influence consumer decisions, highlighting the importance of addressing both positive and negative perceptions in strategies aimed at increasing GMF adoption.

### 2.6. The mediating effect of perceived benefits (PB) and perceived risks (PR)

In addition to examining direct relationships among variables, this study explores how perceived benefits and perceived risks mediate the relationships between the independent variables, knowledge of GMF and trust in GMF, and the dependent variable, purchase intentions. Numerous studies have highlighted the mediating roles of perceived benefits and perceived risks in the context of GMF. For instance, Zhu [38] underscored the critical role of knowledge in shaping consumers’ risk perceptions and their subsequent purchase intentions towards GMF. Knowledge about GMF can alter how individuals perceive potential risks and benefits, thereby influencing their willingness to purchase these products.

Epistemic trust, or trust in the sources of information about GMF, has been identified as a significant precursor to both perceived risks and benefits. It plays an indirect role in affecting GMF acceptance [57]. Trust in the information provided about GMF, including media reports and scientific endorsements, can significantly impact how risks and benefits are perceived, which in turn affects purchase intentions. Research has shown that when media coverage is perceived as biased, individuals tend to view GMF as more hazardous [58]. This highlights how trust in information sources can influence risk perception and, consequently, purchase behavior.

Further supporting the role of perceived benefits and risks, Ghasemi [59] demonstrated that these factors mediate the effects of psychological risk attributes on consumer attitudes and behaviors. For instance, higher perceived health risks associated with GMF can lead to more cautious consumer attitudes, which affect purchase intentions [60]. Conversely, perceived benefits, such as enhanced nutritional value or environmental advantages, can positively influence attitudes and increase the likelihood of purchasing GMF. Lingyu and Liu [45] also found a significant positive correlation between trust in GMF and purchase intention. This suggests that greater trust in GM technology or its production process can enhance purchase intentions, partly by influencing perceived benefits and reducing perceived risks. These findings collectively underscore the importance of understanding the mediating roles of perceived benefits and risks in the relationships between knowledge, trust, and purchase intention. By integrating these mediators into the analysis, a more nuanced understanding of consumer behavior towards GMF can be achieved. Based on these insights, the following hypotheses were proposed:

**H9:** PB significantly mediates the relationship between KN and PI.
**H10:** PB significantly mediates the relationship between TR and PI.
**H11:** PR significantly mediates the relationship between KN and PI.
**H12:** PR significantly mediates the relationship between TR and PI.

### 2.7. Theoretical framework

**Fig 1:**
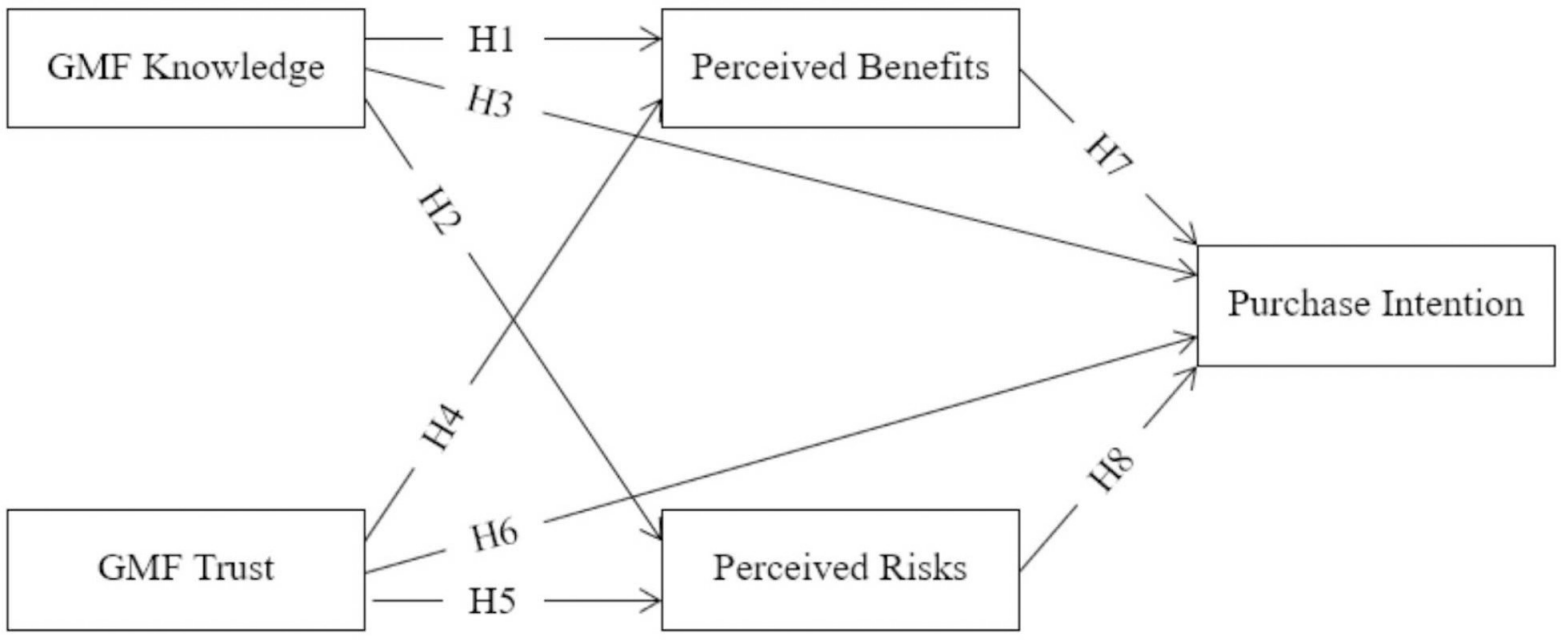
The proposed research framework.

This study focused on evaluating the influence of knowledge and trust on consumer purchase intention, both directly and indirectly through the mediators, perceived benefits and risks. The overarching objective is to conduct a comprehensive analysis aimed at unraveling the intricate dynamics through which knowledge, trust, and perception collectively shape the intention to purchase GMF products among Vietnamese consumers. The interrelationships among the investigated factors and the research framework are represented in Fig 1.

## 3. Methodology

### 3.1. Participants

This study investigates the impact of knowledge and trust on the purchasing intentions of Vietnamese consumers regarding Genetically Modified Foods (GMF), focusing exclusively on individuals aged 18 and above. No data were collected from children, and no experiments involving humans or animals were conducted.

Initially, 647 responses were collected based on participants’ knowledge and familiarity with GMF. After screening out individuals who lacked knowledge about GMF, 424 valid responses were retained. Of the 223 individuals who had never heard of GMF, 43.95% were male and 56.05% were female. Additionally, more than half of the respondents in this group were aged 22 and above. In contrast, among the 424 valid respondents, 157 (37.03%) were male and 267 (62.97%) were female. The majority of participants (353 or 83.25%) were aged 18–21, while 71 participants (16.75%) were aged 22 and above. Regarding the frequency of GMF purchases, 126 individuals (29.72%) rarely bought GMF, 110 (25.94%) purchased GMF only when necessary, 109 (25.71%) were uncertain if they had used GMF, 62 (14.62%) were aware of GMF but had never made a purchase, and 17 (4.01%) consistently bought GMF. These findings illustrate the diverse consumption habits and awareness levels regarding GMF among the surveyed participants.

### 3.2. Questionnaire design

This study employed a quantitative approach, utilizing a 5-point Likert scale questionnaire (1 = Strongly Disagree; 5 = Strongly Agree) as the main tool for collecting primary data. The questionnaire was meticulously designed to gather empirical data for testing the proposed hypotheses and evaluating the research model. To ensure its relevance and accuracy, the questionnaire items were adapted from established studies by Ari [11] and Farid [61], which provided a solid foundation for assessing the constructs within the context of this research. Before its deployment, the questionnaire underwent a thorough review by an expert in consumer behavior study, whose insights were instrumental in refining the instrument. This pre-distribution consultation helped ensure that the questionnaire was both reliable and valid for the study’s objectives.

The questionnaire has four main sections. The initial section provided an overview of the research theme and objectives. It sought participants’ consent to engage in the study, explicitly assuring them that any data collected would be treated with confidentiality and solely used for scientific research purposes. This section was crucial for establishing trust and obtaining informed consent. The second section collected data on participants’ socio-demographic profiles, including gender, age, and occupation. This information was essential for contextualizing the findings and understanding the demographic diversity of the sample. The third section gauged participants’ familiarity with Genetically Modified Foods (GMF) through a screening question. It also explored the frequency of GMF purchases to understand consumer behavior patterns better. This section was pivotal in establishing a baseline for participants’ knowledge and engagement with GMF. The final section of the questionnaire comprised 28 statements designed to measure five distinct constructs: Knowledge (KN), assessed with 4 items, was adapted from Farid [61]; Trust (TR), measured with 7 items, and Perceived Benefits (PB), with 6 items, as well as Perceived Risks (PR), gauged with 7 items, were all adapted from Ari [11] and Farid [61]; and Purchase Intention (PI), with 4 items, was adapted from Ari [11]. Multiple items were used for each construct to ensure a thorough and reliable assessment. Detailed information about these items is presented in S1 Appendix. The selection of these items was based on highly referenced studies published in reputable journals, ensuring that the measures adhered to established standards of validity and reliability.

### 3.3. Data collection

The data collection process was meticulously designed to ensure the relevance and reliability of the findings. The questionnaire was tailored to align with the characteristics of the Vietnamese population. It was originally created in English and translated into Vietnamese, utilizing the back translation method recommended by Brislin [62], to address cultural and language differences. An online survey was conducted via Google Forms and distributed through social media platforms such as Facebook and Zalo to attract a diverse range of participants. All participants provided explicit written consent before engaging in the study. Consent was obtained through a checkbox at the beginning of the survey, confirming their voluntary participation and approval for the use of their information in research. The survey included a clear explanation of the study’s objectives and emphasized the importance of confidentiality and anonymity in handling responses. This study received ethical approval from the Board of Directors at FPT Can Tho University, Vietnam (Approval No. 20231108.04). Ethical guidelines were strictly followed to ensure that all procedures conformed to established standards for research involving human participants, safeguarding participant privacy and ensuring their informed consent throughout the process.

The study employed a non-probability sampling method for data collection, chosen for its inherent advantages in terms of convenience, time efficiency, and cost-effectiveness, as emphasized by Acharya [63]. This inclusive approach aimed to promote participant involvement, thereby facilitating a more comprehensive representation of diverse perspectives. To mitigate potential biases in data collection, participation was voluntary, without monetary incentives, and respondents could opt out without repercussions. The average completion time was about 10 minutes, and participants found the questionnaire language clear and comprehensible.

During November 2023 (from the 9^th^ to the 30^th^), 647 responses were collected. Among these responses, 424 were considered valid and utilized for subsequent analysis. Responses that were incomplete or submitted in under one minute were excluded from the dataset, following the criteria established by Lyu [64]. According to Hair [65], a quantitative research study should have a sample size that is at least five times the total number of observed variables’ items. With 28 observed items, the minimum required sample size was 140 (28 x 5), which was surpassed with 424 respondents. This ensures the precision and reliability of the study’s outcomes.

### 3.4. Data analysis

The primary objective of this research is to assess the measurement and structural models utilizing Partial Least Squares Structural Equation Modeling (PLS-SEM). The systematic application of PLS-SEM aims to scrutinize the relationships between GMF knowledge and trust in the context of purchase intention concerning GMF. This analytical approach enables the identification of direct connections and facilitates the examination of indirect effects through intermediary variables such as Perceived Benefits and Perceived Risks.

Following the two-step approach advocated by Hair [66], data was processed using SmartPLS software version 3.0, incorporating key elements for an in-depth analysis. Descriptive statistics, including outer loadings, mean, and standard deviation, provide a clear data overview. Construct reliability and validity were evaluated using Cronbach’s Alpha, Composite Reliability (CR), and Average Variance Extracted (AVE). These metrics ensure that the constructs are consistently measured and that the items accurately reflect the underlying constructs. Outer loadings were examined to measure the strength of connections between latent variables and their respective indicators. High outer loadings indicate that the indicators are good measures of their respective constructs. Discriminant validity was assessed using the Heterotrait-Monotrait (HTMT) ratio and the Fornell-Larcker criterion. These methods ascertain the distinctiveness of each construct, ensuring that they are not too highly correlated with each other. This step is crucial to confirm that the constructs used in the model are unique and accurately measured. A multicollinearity test was conducted to identify any presence of multicollinearity in the data. This evaluation was based on the variance inflation factor (VIF), which measures how much the variance of an estimated regression coefficient increases due to collinearity. Low VIF values indicate that multicollinearity is not a concern, ensuring the robustness of the regression results. The proposed relationships between variables were examined through hypothesis testing, using parameters such as path coefficient (*β*), standard deviation (STDEV), *t*-statistics, and *p*-values. Path coefficients indicate the strength and direction of the relationships between constructs, while t-statistics and p-values determine the significance of these relationships. Finally, the model’s fit to the observed data was evaluated using R-squared and Q-squared metrics, specifically for the variable Purchase Intention. R-squared measures the proportion of variance in the dependent variable that is predictable from the independent variables, indicating the model’s explanatory power. Q-squared, or predictive relevance, assesses how well the model predicts the data points of the indicators in reflective measurement models.

## 4. Results

This section presents the analysis of the collected data, structured into two key assessments: the measurement model and the structural model. The first part involves an evaluation of the measurement model, presenting descriptive statistics, and assessing the reliability, validity, and discriminant validity of the constructs used in the study. This step ensures that the items used to measure each construct are both consistent and accurate, and that the constructs are distinct from each other. The second part focuses on the structural model, examining the collinearity problem, assessing the explanatory and predictive capabilities of the model and testing the proposed hypotheses.

### 4.1. Measurement model assessment

#### 4.1.1. Descriptive statistics

In this study, a five-point Likert scale was used to assess the extent of agreement among respondents regarding various scale items. related to GMF. The descriptive analysis results, presented in S1 Appendix, provide insights into the participants’ knowledge, trust, perceived benefits, perceived risks, and purchase intentions concerning GMF. The knowledge (KN) items exhibited the highest average mean value of 4.081, with a standard deviation of 0.738, indicating a significant level of awareness and understanding among participants about GMF. This high mean value suggests that the respondents are well-informed about GMF, which is crucial for shaping their perceptions and behaviors. Participants also demonstrated a high level of trust in GMF, as reflected by an average mean value of 3.864 for the trust (TR) items, with a standard deviation of 0.769. This indicates a generally positive attitude towards the safety and reliability of GMF among the respondents, which could influence their acceptance and consumption of GMF products. Moreover, respondents expressed a positive perception of GMF safety and associated benefits, as evidenced by an average mean of 3.780 for perceived benefits (PB) scale items, accompanied by a standard deviation of 0.855. This suggests that participants recognize the potential advantages of GMF, such as enhanced nutritional value and environmental benefits, which contribute to their favorable view of these products. Conversely, the perceived risk (PR) items revealed an average mean of 2.270, with a standard deviation of 0.667. This low mean value implies that GMF is perceived as posing minimal harm to consumers’ health and is viewed favorably in terms of environmental impact. The lower perceived risk suggests that concerns about the negative effects of GMF are not prevalent among the respondents. Lastly, the study explored the purchase intention (PI) variable, which showed an average mean of 3.808 and a standard deviation of 0.898. These metrics indicate a favorable tendency among consumers to purchase GMF products, reflecting a positive correlation between their knowledge, trust, and perceived benefits with their willingness to buy GMF.

The data analysis in S1 Appendix indicated that all scale items were retained, as none exhibited factor loading values less than 0.50, meeting the criteria suggested by Hair [67]. Among the purchase intention items, PI3 displayed the highest factor loading value at 0.887, while the knowledge scale item KN4 recorded the lowest factor loading value at 0.627. Additionally, all items demonstrated significant factor loadings on their latent constructs, evidenced by the P values of all indicators being 0.00 [67]. This underscores the substantial contribution of each observed item to the comprehensive research, confirming the reliability and validity of the measurement model used in this study.

#### 4.1.2. Construct reliability and validity

Reliability and validity are essential facets that demonstrate the robustness of research methodologies and the trustworthiness of research findings [68]. In evaluating the reliability and validity of the data, three crucial criteria were considered: Cronbach’s Alpha, composite reliability (CR), and average variance extracted (AVE).

Cronbach’s Alpha is used as a metric to evaluate the internal consistency or reliability of items, measurements, or ratings within a study [69]. Generally, a Cronbach’s Alpha value of 0.8 or higher indicates an acceptable level of reliability [70], although values exceeding 0.95 may suggest redundancy. In the statistical analysis presented in Table 1, Cronbach’s Alpha scores ranged from 0.783 to 0.893. The highest Cronbach’s Alpha value was observed for Perceived Benefits (PB), while the lowest was associated with Knowledge (KN). Consequently, all indicators with Cronbach’s Alpha values fell within the specified threshold, affirming a sufficient level of internal consistency.

**Table 1:** Construct reliability and validity.

Constructs	Cronbach’s Alpha	CR	AVE
KN	0.783	0.859	0.607
TR	0.872	0.901	0.567
PB	0.893	0.918	0.650
PR	0.880	0.905	0.578
PI	0.871	0.912	0.722

*Note*: KN = Knowledge, TR = Trust, PB = Perceived benefit, PR = Perceived risk, PI = Purchase intention

Composite reliability (CR) is an alternative measure to Cronbach’s Alpha, often utilized alongside structural equation modeling [71]. CR values typically range from 0.70 to 0.95, indicating satisfactory to good reliability [72]. As shown in Table 1, CR values ranged between 0.859 and 0.918, with the PB variable exhibiting the highest value at 0.918. Therefore, the CR scores met the recommended threshold, confirming substantial reliability for all variables.

Average Variance Extracted (AVE) is a metric quantifying the proportion of variance explained by a construct relative to the variance attributed to measurement error [73]. According to Hair [67], it is commonly expected that the AVE for each construct exceeds 0.5 to fulfill the convergent validity criterion. The statistical analysis presented in Table 1 revealed that all AVE values were higher than the recommended value of 0.5, with the PI variable exhibiting the highest value of 0.722. This outcome indicated that all constructs in the study meet the criteria for convergent validity.

In conclusion, the analysis outcomes suggest that the criteria of Cronbach’s Alpha, composite reliability (CR), and average variance extracted (AVE) met the recommended thresholds, affirming the reliability and validity of the data for this study. The high levels of internal consistency, substantial reliability, and convergent validity demonstrate the robustness of the constructs used, ensuring the trustworthiness of the research findings.

#### 4.1.3. Discriminant validity

Discriminant validity refers to the degree to which constructs in a model are distinct and not overlapping, ensuring that each construct is unique and measures a different concept. This validity is crucial for confirming that the constructs are indeed capturing different aspects of the model. In this study, discriminant validity was evaluated using two methods: the Heterotrait-Monotrait (HTMT) ratio and the Fornell-Larcker criterion [74].

The HTMT ratio is defined as the mean value of the correlations between items across different constructs relative to the mean of the average correlations for items measuring the same construct. According to Rasoolimanesh [75], an ideal HTMT value should typically fall below 0.85 or 0.9 to indicate acceptable discriminant validity. As shown in Table 2, the observed HTMT values ranged from 0.142 to 0.884. These values suggest that the constructs used to measure the causal relationships in this study are indeed distinct from one another, thereby supporting the discriminant validity of the model.

**Table 2:** Heterotrait—Monotrait (HTMT) ratios.

Constructs	KN	PB	PI	PR	TR
KN					
PB	0.666				
PI	0.665	0.745			
PR	0.297	0.142	0.260		
TR	0.733	0.884	0.746	0.201	

*Note*: KN = Knowledge, TR = Trust, PB = Perceived benefit, PR = Perceived risk, PI = Purchase intention

The Fornell-Larcker criterion provides another method to assess discriminant validity by comparing the square root of each construct’s AVE with its correlations with other constructs. For discriminant validity to be established, the square root of a construct’s AVE should be greater than its highest correlation with any other construct [76]. Table 3 demonstrates that the square roots of AVE for each construct surpassed their correlations with other constructs. This finding further confirms the discriminant validity among the constructs under consideration.

**Table 3:** Fornell-Larcker criterion.

Constructs	KN	PB	PI	PR	TR
KN	**0.779**				
PB	0.578	**0.806**			
PI	0.567	0.664	**0.850**		
PR	-0.257	-0.123	-0.239	**0.760**	
TR	0.594	0.795	0.657	-0.173	**0.753**

*Note*: KN = Knowledge, TR = Trust, PB = Perceived benefit, PR = Perceived risk, PI = Purchase intention

The measurement model assessment confirmed strong reliability and validity of the constructs. Furthermore, discriminant validity was supported by both the Fornell-Larcker criterion and the HTMT ratio, ensuring that the constructs are distinct. All items met the necessary thresholds, highlighting that the model accurately represents the factors shaping consumer perceptions and purchase intentions toward GMF.

### 4.2. Structural model assessment

In the assessment of the structural model, critical criteria include the path coefficient (*β*), *t*-statistics (*t*), *p*-values (*p*), and the variance inflation factor (VIF). These metrics are vital for understanding the relationships between the constructs and the overall model fit.

#### 4.2.1. Assessment of common method bias

Before evaluating the structural relationships, it is essential to examine collinearity to prevent potential bias in the regression results [77]. The Variance Inflation Factor (VIF) was employed to detect potential linear relationships, or collinearity, among the independent variables in the multiple linear regression model [78]. According to Hair [77], a VIF value of 5 or higher indicates significant collinearity issues among the indicators used for the formative measurement of constructs. In this study, as shown in Table 4, the observed VIF values ranged from 1.077 to 2.952, suggesting the absence of collinearity among predictor variables.

**Table 4:** Hypothesis testing.

H	Structural	Path Coefficient (*β*)	Standard Deviation (STDEV)	*t*-statistics	*p*-values	VIF	Results
H1	KN → PB	0.162	0.041	3.933	0.000	1.546	Accepted
H2	KN → PR	-0.239	0.059	4.035	0.000	1.546	Accepted
H3	KN → PI	0.194	0.050	3.896	0.000	1.692	Accepted
H4	TR → PB	0.699	0.037	19.073	0.000	1.546	Accepted
H5	TR → PR	-0.031	0.080	0.383	0.702	1.546	Rejected
H6	TR → PI	0.257	0.058	4.461	0.000	2.952	Accepted
H7	PB → PI	0.335	0.068	4.950	0.000	2.866	Accepted
H8	PR → PI	-0.104	0.039	2.641	0.008	1.077	Accepted

*Note*: KN = Knowledge, TR = Trust, PB = Perceived benefit, PR = Perceived risk, PI = Purchase intention

#### 4.2.2. Examination of research hypotheses

**Fig 2:**
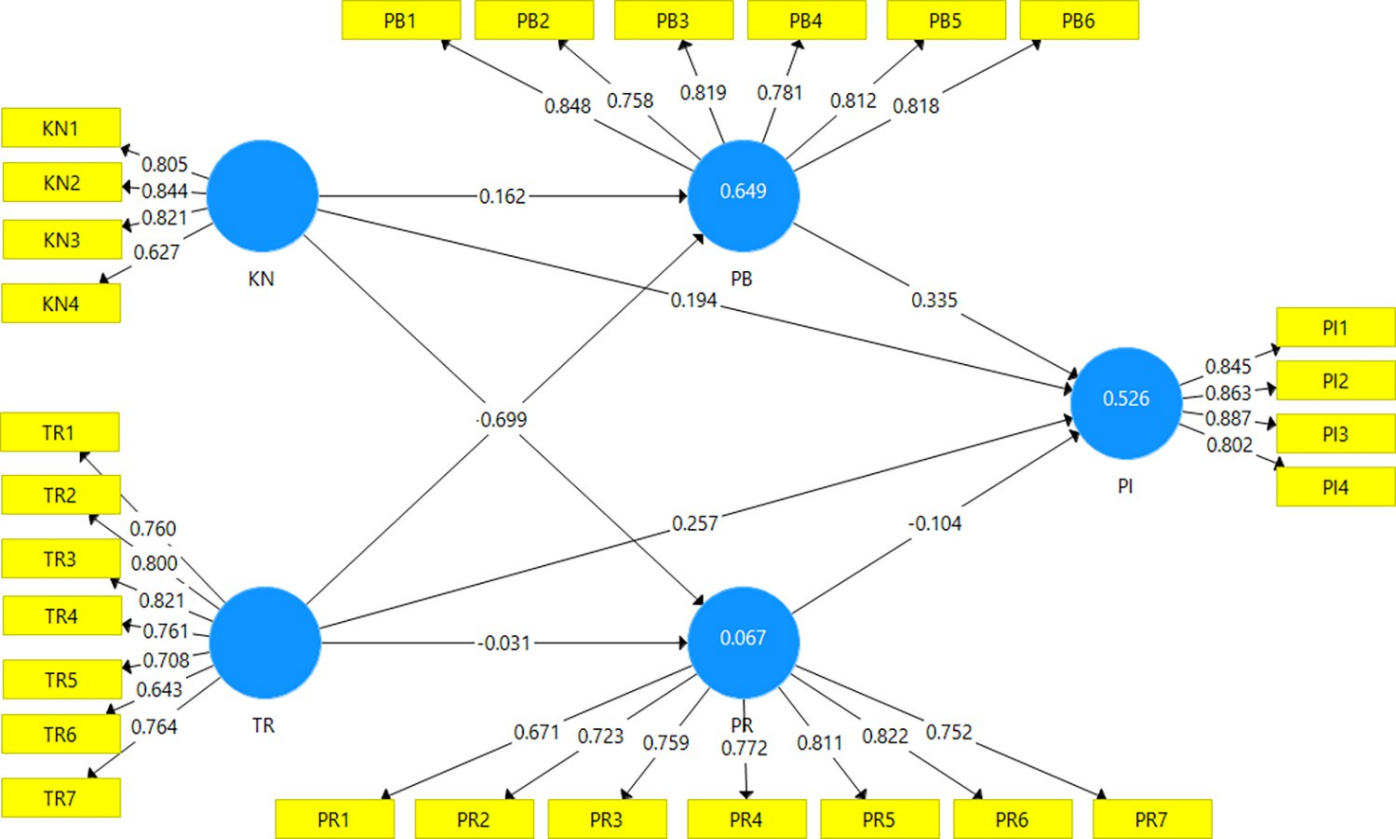
The PLS-SEM results.

Table 4 and Fig 2 present the study outcomes, where most of the proposed hypotheses were supported, except for the correlation between Trust (TR) and Perceived Risk (PR). Specifically, the positive correlation between Knowledge (KN) and Perceived Benefits (PB) was significantly supported (H1: *β* = 0.162, *p* < 0.001). This signifies that as customers enhance their understanding of GMF, they are inclined to believe that its consumption yields greater advantages. Conversely, a negative association between Knowledge (KN) and Perceived Risk (PR) emerged (H2: *β* = -0.239, *p* < 0.001), indicating that deeper comprehension of GMF results in a diminished perception of associated risks. Hypothesis 3 demonstrated a strong positive relationship between Knowledge (KN) and Purchase Intention (PI) (H3: *β* = 0.194, *p* < 0.001). This indicated that as customers acquire more insight into GMF, their intention to make a purchase increases. Similarly, Trust (TR) exhibited a positive impact on Perceived Benefits (PB) (H4: *β* = 0.699, *p* < 0.001). This reflected that when customers put greater trust in GMF products, they perceive these items as having more benefits than other product types. However, the anticipated negative relationship between Trust (TR) and Perceived Risk (PR) was not supported, as the p-value exceeded the acceptable threshold (H5: *β* = -0.031, *p* > 0.05). In other words, an increase in customer trust does not significantly influence their perception of risks. Nevertheless, Trust (TR) still had a direct positive effect on Purchase Intention (PI) (H6: *β* = 0.257, *p* < 0.01). This implied that as customer trust in change positively progresses, their intentions to purchase GMF increase. Furthermore, the study revealed a positive relationship between Perceived Benefits (PB) and Purchase Intention (PI) (H7: *β* = 0.335, *p* < 0.001). This implied that customers who believe GMF offers health and related benefits have a greater tendency to exhibit an intention to consume it. Lastly, the relationship between Perceived Risk (PR) and Purchase Intention (PI) was confirmed (H8: *β* = -0.104, *p* < 0.05), indicating that an increase in perceived risks decreases the intention to purchase. The results suggested a complex interaction of knowledge, trust, perceived benefits, and risks influencing consumer intentions towards GMF consumption.

R-squared and Q-squared values are metrics used to assess the explanatory and predictive capabilities of the model. In this study, the R-squared value for PI was 0.526, indicating a moderate explanatory power [79]. This value suggested that approximately 52.6% of the variance in purchase intention is accounted for by the variance between perceived benefits and perceived risks. Another metric for evaluating predictive accuracy, Q-squared values, are considered satisfactory when greater than zero for a specific endogenous construct to indicate the predictive accuracy of the structural model for that construct [77]. In this study, the Q-squared value for PI was reported as 0.376, indicating a medium level of predictive relevance for the PLS-path model [77]. These values collectively suggest the model’s capacity to predict consumer purchase intention with an accuracy of around 37.6%. Therefore, the R-squared and Q-squared values demonstrate the model’s ability to moderately explain and predict consumer purchase intention based on perceived benefits and risks.

In the analysis results presented in Table 5, three out of four hypotheses concerning indirect effects were confirmed, except for the mediating role of Perceived Risk (PR) between Trust (TR) and Purchase Intention (PI). Specifically, concerning the mediating role of Perceived Benefit (PB), the results indicated that Perceived Benefit (PB) significantly mediates the relationship between Knowledge (KN) and Purchase Intention (PI), which was supported (H9: *β* = 0.054, *p* < 0.05). This implies that as consumers’ knowledge increases, their perceived benefits tend to rise, consequently promoting their intention to purchase. Similarly, Trust (TR) has a significant impact on Purchase Intention (PI) through the Perceived Benefit (PB) mediator, as confirmed (H10: *β* = 0.234, *p* < 0.001). This result suggests that improvement in customer trust positively influences perceived benefits, subsequently raising purchase intention. Regarding the mediating role of Perceived Risk (PR), Knowledge (KN) demonstrated a significant indirect influence on Purchase Intention (PI) through Perceived Risk (PR) (H11: *β* = 0.025, *p* < 0.05). As consumers acquire more knowledge about GMF, their perception of risks diminishes, thereby stimulating a propensity to consume. However, the connection between Trust (TR) and Purchase Intention (PI) through the mediating role of Perceived Risk (PR) was not supported (H12: *β* = 0.003, *p* > 0.05), as the p-value exceeded 0.05.

**Table 5:** Indirect effects.

H	Structural	Path Coefficient (*β*)	Standard Deviation (STDEV)	*t*-statistics	*p*-values	Results
H9	KN → PB → PI	0.054	0.017	3.107	0.002	Accepted
H10	TR → PB → PI	0.234	0.051	4.594	0.000	Accepted
H11	KN → PR → PI	0.025	0.012	2.130	0.033	Accepted
H12	TR → PR → PI	0.003	0.009	0.345	0.730	Rejected

*Note*: KN = Knowledge, TR = Trust, PB = Perceived benefit, PR = Perceived risk, PI = Purchase intention

In summary, the model demonstrated a moderate ability to explain and predict consumer purchase intention. The structural model assessment showed that most hypotheses are supported, indicating significant relationships between knowledge, trust, perceived benefits, perceived risks, and purchase intention towards GMF, except for the correlation between trust and perceived risk. The analysis of indirect effects revealed that perceived benefits and risks mediate the relationship between knowledge and purchase intention. However, while perceived benefits played a mediating role between trust and purchase intention, perceived risk does not significantly mediate this relationship.

## 5. Discussion

This study investigates the influences of knowledge and trust on shaping perceptions and purchase intentions towards genetically modified foods (GMF) among Vietnamese consumers, employing Partial Least Squares Structural Equation Modeling (PLS-SEM) statistical analysis. The findings provide substantial support for most hypotheses, highlighting significant impacts of knowledge and trust on consumer perceptions of GMF benefits and risks, as well as their purchase intentions.

Firstly, the research underscores the pivotal role of knowledge in influencing consumer perceptions and purchase intentions regarding GMF. Specifically, it identifies a positive relationship between knowledge (KN) and perceived benefits (PB), alongside a negative association between knowledge (KN) and perceived risks (PR). This suggests that as consumers gain a deeper understanding of GM-related food technologies, they are more likely to perceive GMF as contributing positively to societal and economic well-being, while simultaneously reducing concerns about associated risks. These findings are consistent with prior research. For instance, Zakaria [80] explored the perspective of farmers, revealing that knowledge about GM crops significantly influences their perception of GMO technology in commercial agricultural production. In addition, Raza [35] observed that heightened knowledge can mitigate perceptions of GMF risks, particularly regarding health hazards. Besides, Ari [11] found that increased consumer knowledge enhances perceptions of benefits derived from gene technology in food production. Thus, enhancing consumer knowledge through targeted educational efforts could foster more positive perceptions and attitudes towards GMF. Moreover, the study reveals a direct positive impact of knowledge (KN) on purchase intention (PI). This correlation is logical, as consumers with a better understanding of GMF are more likely to exhibit stronger intentions to purchase these products. This finding aligns with previous research indicating that increased consumer knowledge correlates positively with purchase intentions [13, 38]. For instance, Zhu [38] indicated that as consumers acquire greater knowledge, their inclination to make a purchase increase. Hwang and Nam [13] also identified that consumers with higher levels of both objective and subjective knowledge tend to exhibit stronger purchase intentions towards GMF. These findings underscore the significant role of knowledge in not only shaping consumer perceptions but also influencing their purchasing behavior. As consumers deepen their understanding, they increasingly view GMF as beneficial contributors to society and the economy, thereby reducing concerns about associated risks. These findings offer strategic recommendations for businesses to capitalize on opportunities through targeted marketing campaigns aimed at fostering positive consumer perceptions, attitudes, and behaviors towards GMF. Marketers should prioritize communicating the advantages of GM products, such as their enriched nutritional content and potential to address global food challenges, to enhance consumers’ knowledge and perceived benefits regarding GMF. This approach aims to cultivate a more favorable perception among consumers. Furthermore, businesses should strategically invest in GMF-related products, with a focus on conveying essential information through labels. This aspect is crucial as labels significantly influence both consumer perceptions and purchase intentions. By leveraging these strategies, businesses can effectively communicate the value of GMF and enhance their credibility within the marketplace.

Secondly, the study further elucidates the influence of trust (TR) on perceptions and purchase intentions towards GMF. It identifies a positive correlation between trust (TR) and perceived benefits (PB), indicating that consumers who trust GM products are more likely to perceive them as having greater advantages. This finding aligns with previous research by Chen and Li [81] and Ari [11], affirming that trust in scientists, government entities, labeling systems, and the media can increase perceived benefits associated with GMF. However, contrary to expectations, the study did not find a significant negative correlation between trust (TR) and perceived risks (PR). This suggests that while trust in GM products may enhance perceived benefits, it does not necessarily alleviate concerns about potential risks associated with GMF. This finding diverges from Hu [57], who suggested that higher trust in industrial organizations correlates with a reduction in perceived risks. Cultural factors and the nuanced perceptions of Vietnamese consumers may contribute to this discrepancy, indicating a need for context-specific strategies to address trust and risk perceptions effectively. Nonetheless, the study confirms a direct positive influence of trust (TR) on purchase intention (PI). Consumers who trust GMF products are more inclined to purchase and consume them, believing in their safety, reliability, and alignment with their expectations. This finding is supported by research indicating that trust serves as a significant predictor of consumer acceptance and purchase intentions towards GMF [44, 47]. When consumers place trust in the technology or the production process of GMF, they demonstrate positive intentions towards purchasing these products [46]. Hence, trust emerges as a crucial determinant of purchase intention in the Vietnam market. As industries develop, understanding the complex relationships between trust, perceived benefits, perceived risks, and purchase intention becomes imperative for effectively communicating with and attracting consumers, especially in the economic landscape of GM products. Therefore, fostering consumer trust through transparent communication and assurances of safety could enhance market acceptance and consumer adoption of GMF products in Vietnam.

Thirdly, the study identifies significant correlations between consumer perceptions and purchase intentions towards GMF. It found a positive relationship between perceived benefits (PB) and purchase intention (PI), indicating that consumers who perceive greater advantages associated with GMF are more likely to purchase these products. Conversely, a negative correlation is observed between perceived risks (PR) and purchase intention (PI), suggesting that heightened concerns about risks diminish consumers’ intentions to consume GMF. These findings are consistent with prior studies emphasizing the critical roles of perceived benefits and risks in shaping consumer acceptance and purchase behaviors towards GMF [53, 81]. In other words, individuals with high perceptions of benefits are more likely to purchase GMF, while those perceiving greater risks are less inclined to do so. This consolidated evidence underscores the critical importance of the complex relationship between perceived risks and benefits in shaping consumer purchase intentions in the context of GM products.

Moreover, the study explores the mediating roles of perceived benefits (PB) and perceived risks (PR) between knowledge (KN), trust (TR), and purchase intention (PI). Building upon prior research such as Choi and Kim [82], which explored the indirect association between objective knowledge and organic food purchases through increased risk perception, this study extends this understanding by introducing perceived benefits as a crucial mediating factor. Specifically, this study’s findings reveal a correlation between knowledge, trust, and purchase intention of GMF mediated by perceived benefits. This suggests that deeper knowledge and greater trust in GMF lead to enhanced perceptions of benefits among consumers, thereby fostering purchase intention. Additionally, this study uncovered the mediating role of perceived risks between knowledge and purchase intention, indicating that increased knowledge positively influences purchase intention by reducing perceived risks. Partially aligning with Hu [57], who emphasized the significance of epistemic trust in GMF acceptance by directly influencing perceived benefits and risks, our research emphasizes the pivotal role of perceived benefits and risks in mediating the relationship between epistemic trust and acceptance. However, unlike their findings, this study did not observe the mediating role of perceived risks in the relationship between trust and purchase intention. This disparity may stem from regional or contextual differences in consumer behavior. It underscores the importance of considering the unique characteristics and preferences of consumers in different areas. Overall, the mediating roles of perceived benefits and risks offer valuable insights into the mechanisms shaping customers’ purchase intentions and shed light on the multifaceted nature of knowledge and trust in the context of GMF.

In conclusion, this study provides comprehensive insights into the intricate relationships between knowledge, trust, perceived benefits, perceived risks, and purchase intentions among Vietnamese consumers towards GMF. The findings emphasize the importance of enhancing consumer knowledge and trust through targeted educational campaigns and transparent communication strategies. These efforts can help mitigate perceived risks and enhance perceived benefits associated with GMF, thereby fostering consumer acceptance and purchase intentions. Marketers and policymakers should tailor their strategies to address the unique cultural and perceptual dynamics influencing consumer behaviors in Vietnam, thereby promoting the sustainable adoption of GMF products in the marketplace.

### 5.1. Limitations and recommendations

This study acknowledges several limitations that need to be considered. Firstly, the research is limited by a small sample size and an age imbalance, with a skew towards younger individuals due to the convenience sampling method used in online data collection. These factors may affect the generalizability and representativeness of the findings. To improve external validity, future studies should prioritize larger, more diverse samples that include individuals from various generations, regions, socio-economic backgrounds, and age groups, ensuring a more balanced and comprehensive representation of consumer segments. Secondly, the use of self-report methods is susceptible to personal biases, potentially leading to skewed results. To mitigate this influence, future research could employ a variety of data collection methods, including observational studies, experiments, or qualitative interviews. These approaches would offer complementary insights and help triangulate findings, ensuring a more robust understanding of consumer perceptions and behaviors towards GMF. Thirdly, the omission of an analysis of demographic factors such as occupation, education, and income level may limit the comprehensiveness of the study. These factors can significantly influence consumer perceptions and behaviors. Therefore, future research should include an in-depth examination of demographic variables to provide nuanced insights into how socio-economic characteristics shape attitudes and intentions towards GMF.

### 5.2. Implications

This research contributes to both theoretical advancements and practical applications in the domain of genetically modified foods (GMF). Theoretically, it enhances the existing literature by elucidating the roles of knowledge and trust in shaping consumer perceptions and purchase intentions of GMF. The study establishes a clear linkage between the acquisition of knowledge, development of trust, and their combined impact on consumer attitudes towards GMF products. Furthermore, it extends theoretical frameworks by proposing a model where knowledge and trust influence perceptions of benefits and risks, thereby affecting consumer purchase intentions. Moreover, this study provides valuable insights into the influence of cultural factors on consumer attitudes in Vietnam. Understanding these dynamics contributes to a deeper understanding of cross-cultural variations in consumer perceptions of GMF, laying the groundwork for future comparative studies across different cultural contexts.

Practically, the findings offer actionable recommendations for businesses and policymakers involved in GMF marketing and regulation. The study advocates for strategic marketing approaches that emphasize the positive relationship between knowledge and favorable perceptions of GMF benefits. By promoting educational campaigns that highlight the nutritional advantages and societal benefits of GMF, businesses can potentially enhance consumer acceptance and uptake of GMF products. Additionally, the study underscores the importance of building and maintaining trust among various stakeholders involved in GMF production and regulation. Strengthening trust in scientists, government bodies, labeling systems, and media can foster positive perceptions of GMF safety and reliability among consumers. This is particularly crucial given the study’s findings that trust positively correlates with perceived benefits of GMF. Furthermore, addressing and mitigating perceived risks associated with GMF through transparent communication and effective risk management strategies is essential. Businesses should adopt nuanced communication approaches that acknowledge consumer concerns while emphasizing the rigorous safety assessments and regulatory frameworks governing GMF products.

In conclusion, this study highlights the ongoing need for businesses and policymakers to educate consumers about the safety, nutritional benefits, and societal contributions of GMF. By aligning marketing strategies with consumer knowledge and trust-building efforts, stakeholders can cultivate a favorable environment for GMF acceptance and adoption in Vietnam and potentially in other similar markets globally.

## 6. Conclusion

This study has explored the intricate dynamics influencing consumers’ intentions to purchase genetically modified foods (GMF) in Vietnam, focusing on the roles of knowledge and trust while considering perceived benefits and risks as mediating factors. The findings underscore the multifaceted nature of consumer decision-making in the context of GMF.

The empirical analysis provides robust support for most hypotheses, revealing direct and indirect pathways through which knowledge and trust influence purchase intentions. Specifically, heightened knowledge about GMF correlates positively with perceptions of increased benefits and decreased risks. This suggests that as consumers become more informed about GMF, they tend to view these products favorably in terms of societal and economic contributions while mitigating concerns about potential adverse effects. Moreover, the study demonstrates that trust in GMF enhances perceptions of their benefits without significantly altering perceptions of associated risks. This nuanced finding suggests that while trust influences positive attitudes towards GMF, it may not entirely alleviate consumer concerns about safety and environmental impact. Importantly, perceived benefits emerge as a crucial driver of purchase intentions. Consumers who perceive GMF as offering nutritional advantages and societal benefits exhibit a higher propensity to purchase these products. Conversely, heightened perceptions of risks associated with GMF dampen consumer intentions to buy them.

These findings highlight the complex interplay among knowledge, trust, perceived benefits, and perceived risks in shaping consumer behaviors towards GMF. They underscore the importance of targeted communication strategies that emphasize the benefits of GMF while addressing consumer concerns transparently. Such strategies could enhance consumer trust and positively influence purchase intentions.

This study contributes valuable insights into consumer behavior in Vietnam’s GMF market, offering theoretical advancements and practical implications for businesses and policymakers. By understanding the drivers of consumer perceptions and purchase intentions, stakeholders can tailor their strategies to foster acceptance and uptake of GMF products, thereby contributing to sustainable agricultural practices and addressing global food security challenges. Continued research in this area is essential to further refine strategies and adapt to evolving consumer attitudes and preferences towards GMF.

## Supporting information

S1 Appendix Descriptive statistics. (DOCX)

S1 File Survey questionnaire. (PDF)

S2 File Dataset used in analysis. (XLSX)
