# Supplementary material for: Impacts of knowledge and trust on consumer perceptions and purchase intentions towards genetically modified foods
Source: PLoS One. 2024 Oct 2;19(10):e0311257. doi: 10.1371/journal.pone.0311257 (PMC11446447; doi:10.1371/journal.pone.0311257)
Supplement: S1 Appendix — (DOCX) [file pone.0311257.s001.docx]

# Appendix.

# Descriptive Statistics

| **Construct** | **Items** | **Outer loadings** | **Mean** | **Standard Deviation** |
| --- | --- | --- | --- | --- |
| Knowledge | KN1: “I understand what GMF is.” | 0.805 | 4.167 | 0.757 |
|  | KN2: “I understand the potential of utilizing GM technology on crops/foods.” | 0.844 | 4.132 | 0.705 |
|  | KN3: “I understand the potential of utilizing GM technology on human health.” | 0.821 | 3.896 | 0.658 |
|  | KN4: “I am willing to learn more about GM technology.” | 0.627 | 4.130 | 0.830 |
|  | **Average** |  | **4.081** | **0.738** |
| Trust | TR1: “I trust the studies and reports of scientists on GMF.” | 0.760 | 3.998 | 0.805 |
|  | TR2: “I have trust in biotech companies that aim to utilize GM technology.” | 0.800 | 3.828 | 0.756 |
|  | TR3: “I have trust in the farmers who utilize GM farming techniques.” | 0.821 | 3.776 | 0.739 |
|  | TR4: “I trust the labeling systems for consumers to recognize GMF.” | 0.761 | 3.781 | 0.760 |
|  | TR5: “I trust that the government controls the use of genetic modification technology in products rigorously.” | 0.708 | 3.892 | 0.772 |
|  | TR6: “I have trust that biotechnology is providing great value to society.” | 0.643 | 4.193 | 0.711 |
|  | TR7: “I trust the publications of the media (newspapers, magazines, TV, etc.) about GMF.” | 0.764 | 3.580 | 0.837 |
|  | **Average** |  | **3.864** | **0.769** |
| Perceived Benefits | PB1: “I find it appropriate to use GMOs to extend the shelf life of the products.” | 0.848 | 3.781 | 0.867 |
|  | PB2: “I find it appropriate to use GMOs to produce products that are more resistant to agricultural diseases and pests.” | 0.758 | 3.943 | 0.856 |
|  | PB3: “I think that genetically modified food shall bring more health benefits for its consumers.” | 0.819 | 3.715 | 0.790 |
|  | PB4: “I think that genetically modified food shall be financially suitable for the majority.” | 0.781 | 3.634 | 0.904 |
|  | PB5: “I think that genetically modified food will help reduce hunger in developing countries.” | 0.812 | 3.682 | 0.911 |
|  | PB6: “Utilizing gene editing technology shall enhance the nation’s economy and society.” | 0.818 | 3.925 | 0.800 |
|  | **Average** |  | **3.780** | **0.855** |
| Perceived Risks | PR1: “The use of gene technology in food production causes environmental problems.” | 0.671 | 2.243 | 0.579 |
|  | PR2: “GMOs are risky for all living things in nature.” | 0.723 | 2.295 | 0.670 |
|  | PR3: “I think that if I eat GMF, it will create a negative effect on my health.” | 0.759 | 2.349 | 0.687 |
|  | PR4: “I think that If I eat GMF, my genome might get affected.” | 0.772 | 2.323 | 0.695 |
|  | PR5: “I think that If I consume genetically edited products, it may negatively affect my descendants.” | 0.811 | 2.250 | 0.672 |
|  | PR6: “I think that utilizing GMF might create more allergies.” | 0.822 | 2.252 | 0.687 |
|  | PR7: “I think the risks of using GMF are still unclear.” | 0.752 | 2.177 | 0.676 |
|  | **Average** |  | **2.270** | **0.667** |
| Purchase Intention | PI1: “If there were GMF in the department stores, my frequency of purchase would be.” | 0.845 | 3.712 | 0.837 |
|  | PI2: “If GMF were sold by the farmers, my frequency of purchase would be.” | 0.863 | 3.790 | 0.793 |
|  | PI3: “If promotional items were offered along with GMF, my frequency of purchase would be.” | 0.887 | 3.821 | 0.940 |
|  | PI4: “If GMF were sold, my frequency of preference compared to normal foods would be.” | 0.802 | 3.908 | 1.020 |
|  | **Average** |  | **3.808** | **0.898** |
