# Supplementary material for: Impacts of knowledge and trust on consumer perceptions and purchase intentions towards genetically modified foods
Source: PLoS One. 2024 Oct 2;19(10):e0311257. doi: 10.1371/journal.pone.0311257 (PMC11446447; doi:10.1371/journal.pone.0311257)
Supplement: S1 File — (PDF) [file pone.0311257.s002.pdf]

QUESTIONNAIRE

IMPACTS OF KNOWLEDGE AND TRUST ON CONSUMER PERCEPTIONS AND PURCHASE INTENTIONS TOWARDS GENETICALLY MODIFIED FOODS

Dear Sir/ Madam,

We are a research team from FPT Can Tho University conducting a study entitled ***"Impacts of Knowledge and Trust on Consumer Perceptions and Purchase Intentions towards Genetically Modified Foods."*** Our goal is to gain a deeper understanding of consumer views on genetically modified foods (GMF) and to explore how knowledge and trust influence perceptions and purchase intentions regarding GMF.

We greatly value your participation in this survey, as your insights are crucial to the success of our research. Please be assured that all information you provide will be kept confidential and used solely for research purposes.

Thank you for your support and contribution to this study.

I. Do you consent to participate in this study and permit us to use the information you provide for research purposes?

☐ Yes

☐ No

II. Personal Information

1. Gender
   ☐ Male
   ☐ Female

2. Age
   ☐ From 18 to 21
   ☐ From 22 to 26
   ☐ From 27 to 30
   ☐ Over 30

3. Occupation
   ☐ Student
   ☐ Business

☐ Staff Office
☐ Other:______

III. Awareness level

GMF are foods produced from or using genetically modified organisms that have had their genetic material (DNA) altered in a way that does not occur naturally or in natural recombination (World Health Organization, 2014).

1. Are you familiar with GMF?
   ☐ Yes (continue)
   ☐ No (stop)

2. How often do you purchase GMF?
   ☐ I am aware, but I have never made purchases
   ☐ I rarely make purchases
   ☐ I purchase when necessary
   ☐ I regularly make purchases
   ☐ I am not sure whether I have used it before or not

IV. Factors influencing consumer purchase intentions towards GMF

Please check (✓) and honestly assess yourself based on your actual behavior given the statements, using the following scales:

1 - Strongly Disagree 2 - Disagree 3 - Neutral 4 - Agree 5 - Strongly Agree

**A. Knowledge of GMF**	**1**	**2**	**3**	**4**	**5**
1. I understand what GMF is.					
2. I understand the potential of utilizing GM technology on crops/foods.					
3. I understand the potential of utilizing GM technology on human health.					
4. I am willing to learn more about GM technology.					

B. Trust on GMF	1	2	3	4	5
1. I trust the studies and reports of scientists on GMF.					
2. I have trust in biotech companies that aim to utilize GM technology.					
3. I have trust in the farmers who utilize GM farming techniques.					
4. I trust the labeling systems for consumers to recognize GMF.					
5. I trust that the government controls the use of genetic modification technology in products rigorously.					
6. I have trust that biotechnology is providing great value to society.					
7. I trust the publications of the media (newspapers, magazines, TV, etc.) about GMF.					
**C. Perceived Benefits**	**1**	**2**	**3**	**4**	**5**
1. I find it appropriate to use GMOs to extend the shelf life of the products.					
2. I find it appropriate to use GMOs to produce products that are more resistant to agricultural diseases and pests.					
3. I think that genetically modified food shall bring more health benefits for its consumers.					
4. I think that genetically modified food shall be financially suitable for the majority.					
5. I think that genetically modified food will help reduce hunger in developing countries.					
6. Utilizing gene editing technology shall enhance the nation’s economy and society.					
**D. Perceived Risks**	**1**	**2**	**3**	**4**	**5**

1. The use of gene technology in food production causes environmental problems.					
2. GMOs are risky for all living things in nature.					
3. I think that if I eat GMF, it will create a negative effect on my health.					
4. I think that If I eat GMF, my genome might get affected.					
5. I think that If I consume genetically edited products, it may negatively affect my descendants.					
6. I think that utilizing GMF might create more allergies.					
7. I think the risks of using GMF are still unclear.					
**E. Purchase Intention**	**1**	**2**	**3**	**4**	**5**
1. If there were GMF in the department stores, my frequency of purchase would be.					
2. If GMF were sold by the farmers, my frequency of purchase would be.					
3. If promotional items were offered along with GMF, my frequency of purchase would be.					
4. If GMF were sold, my frequency of preference compared to normal foods would be.					

Thank you once again for your support!

Thi Thuy An Ngo
Researcher

Thi Yen Nhi Phan
Researcher

Thi Ngoc Trang Le
Researcher
